# Renal Hypoplasia and Oligomeganephronia in a Fetus with Wolf–Hirschhorn Syndrome

**DOI:** 10.3390/diagnostics15212687

**Published:** 2025-10-24

**Authors:** Maria Paola Bonasoni, Mariangela Pati, Khush Shah, Andrea Musarò, Immacolata Blasi, Flavio Vanacore, Giovanna Botticelli, Veronica Barbieri, Veronica Bizzarri, Maria Marinelli, Moira Foroni, Lorenzo Aguzzoli, Marzia Pollazzon

**Affiliations:** 1 Pathology Unit, Azienda USL-IRCCS di Reggio Emilia, 42122 Reggio Emilia, Italy; moira.foroni@ausl.re.it; 2 Unit of Obstetrics and Gynecologic Oncology, Azienda USL-IRCCS di Reggio Emilia, 42122 Reggio Emilia, Italy; mariangela.pati@ausl.re.it (M.P.); andrea.musaro@ausl.re.it (A.M.); immacolata.blasi@ausl.re.it (I.B.); flavio.vanacore@ausl.re.it (F.V.); giovanna.botticelli@ausl.re.it (G.B.); lorenzo.aguzzoli@ausl.re.it (L.A.); 3 Department of Medicine, Lake Erie College of Osteopathic Medicine at Seton Hill, Greensburg, PA 15601, USA; ksshah0318@gmail.com; 4 Medical Genetics Unit, Azienda USL-IRCCS di Reggio Emilia, 42122 Reggio Emilia, Italy; veronica.barbieri2@ausl.re.it (V.B.); veronica.bizzarri@ausl.re.it (V.B.); maria.marinelli@ausl.re.it (M.M.); marzia.pollazzon@ausl.re.it (M.P.)

**Keywords:** Wolf–Hirschhorn syndrome (WHS), renal hypoplasia, oligomeganephronia

## Abstract

**Background and Clinical Significance**: Wolf–Hirschhorn syndrome (WHS, OMIM #194190) is caused by deletion of the distal short arm of chromosome 4. It is characterized by intrauterine growth restriction (IUGR), developmental delay, epilepsy, distinctive facial features, and urinary tract anomalies, particularly renal hypoplasia. However, the histological profile of renal involvement in WHS is rarely documented. **Case presentation**: We report a case of fetal WHS with renal hypoplasia and histological evidence of oligomeganephronia (OMN). At 21 weeks’ gestation, a prenatal ultrasound revealed oligo/anhydramnios and IUGR. Genetic testing (karyotype and CGH-array) confirmed a de novo 17.92 Mb terminal deletion from 4p16.3 to 4p15.31. The pregnancy was legally terminated at 23 weeks. The autopsy showed characteristic WHS dysmorphisms, growth restriction, and markedly small kidneys. Histology revealed OMN with a thinned renal cortex with reduced glomeruli, mainly hypoplastic, some of which were hypertrophic, and dilated proximal tubules. Scattered medullary tubules were present within the tubulointerstitial compartment, alongside thickened tubular basement membranes highlighted by Collagen IV staining. **Conclusions**: This case suggests that OMN may be a histological hallmark of renal hypoplasia in WHS, especially in larger 4p deletions. Recognizing this pattern may help with prenatal prognosis and clinical management. Further studies are needed to confirm this association.

## 1. Introduction

Wolf–Hirschhorn syndrome (WHS, OMIM #194190) is a congenital malformation syndrome due to the deletion of the distal part of the short arm of chromosome 4. For this condition, WHS is also defined as the 4p syndrome or 4p deletion syndrome [1].

WHS presents a prevalence of 1:20,000–50,000 births and a female-to-male ratio of 2:1 [2]. It is characterized by a distinct phenotype that includes intrauterine growth restriction (IUGR), followed by postnatal short stature, low body weight, hypotonia, intellectual disability, epilepsy, and typical craniofacial features described as “Greek warrior helmet.” The craniofacial features include a broad nasal bridge extending to the forehead, a high anterior hairline with a prominent glabella, highly arched eyebrows, wide-set eyes, epicantic folds, a short philtrum, downturned corners of the mouth, and microretrognathia [3]. The ears can be set low or rotated posteriorly, and associated pits, tags, or lobes may be absent. The shape alteration is due to altered or deficient cartilage growth. Other anomalies affect the cardiovascular system, the genitourinary tract, and the central nervous system, resulting in microcephaly, and eye and inner ear deficiencies [3].

The genetic defect typically involves a partial deletion of the distal short arm of chromosome 4.

The minimal critical region (MCR) for WHS is situated about 2 Mb from the 4p telomere, within the 4p16.3 band. This region, spanning approximately 200–750 kilobases (kb), is gene-rich and typically includes deletions of genes such as *WHSC1*, *LETM1*, *NSD2*, *CPLX1*, and *PIGG* [4,5].

The extent of the deletion may vary from 2 Megabases (Mb) up to 30 Mb, being associated with a phenotypic variability. A deletion of less than 3.5 Mb exhibits mild clinical abnormalities confined to peculiar facial dysmorphism, growth retardation, mild intellectual disability, and seizures [6]. A deletion between 5 and 18 Mb is the most common, and those affected exhibit severe growth retardation, hypotonia, important neurodevelopmental dysfunction, and major congenital malformations [6]. The largest deletion (22–25 Mb) is associated with a severe neurological phenotype, which is hardly attributable to WHS [6].

The WHS phenotype can also result from complex chromosomal rearrangements, such as translocations or ring chromosomes. These unbalanced translocations may occur de novo or be inherited from a parent carrying a balanced chromosomal rearrangement.

The most commonly detected translocations are as follows: t(4p;8p); t(4p;7p); t(4p;11p); t(4p;20q); t(4p;21q); and t(4p;12p). Other chromosomal anomalies may include inverted duplications with terminal deletions on the same 4p arm or unbalanced pericentric inversions [3,6].

The prenatal phenotype of WHS varies according to the extent of 4p deletion, and ultrasound (US) findings are usually aspecific, with IUGR being the most common feature. Conversely, facial features (hypertelorism, highly arched eyebrows, long philtrum, and microretrognathia) are difficult to see in utero, but when present, they may raise suspicion for the syndrome. Additional malformations may be detected, including the following: microcephaly, brain abnormalities, a hypoplastic nasal bone, a cleft lip and palate, congenital heart defects, and skeletal anomalies. The urinary tract is commonly affected with renal hypoplasia, ectopic and/or hyperechogenic kidneys, and hydronephrosis [1,7].

Renal hypoplasia is a recognized anomaly in WHS; however, its histological features have rarely been mentioned. Only in five fetuses and four pediatric cases has oligomeganephronia (OMN) been described as the causative microscopic abnormality [8,9,10,11].

Herein, we present a case of fetal WHS with a prenatal diagnosis of 4p deletion, with oligo/anhydramnios at US, severe renal hypoplasia at postmortem, and OMN at histological examination.

## 2. Case Presentation

### 2.1. Clinical History

**Figure 1:**
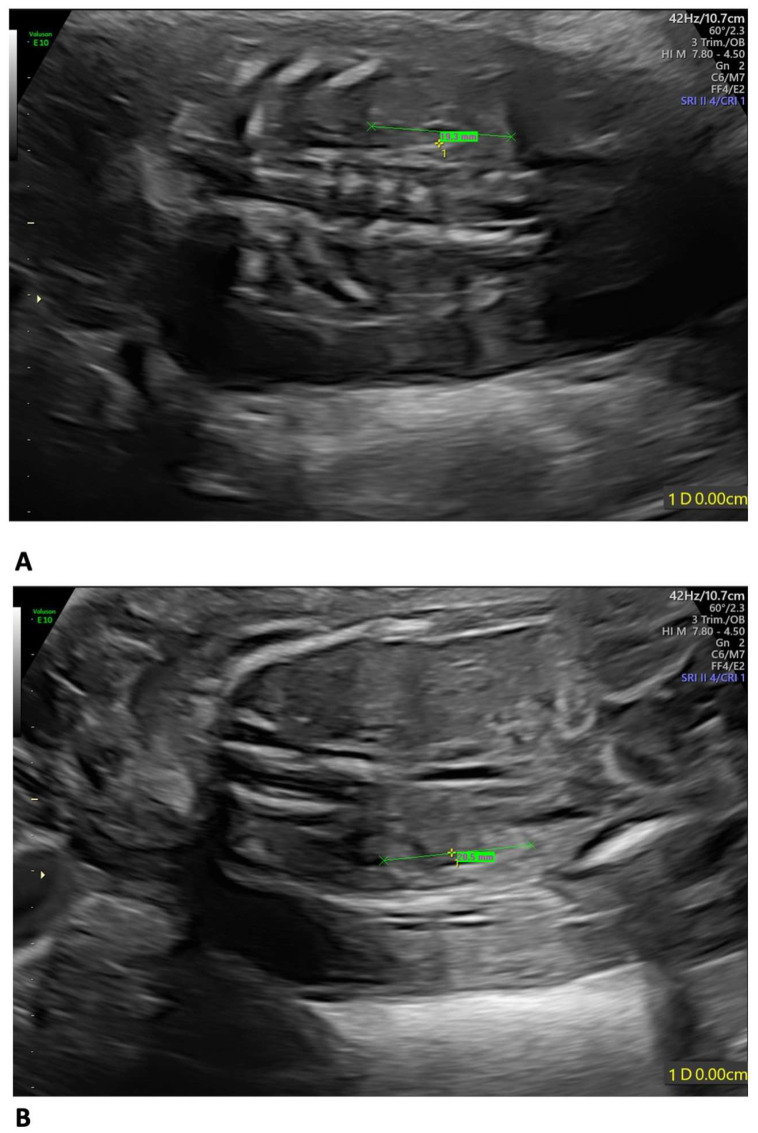
Fetal US at 21 weeks + 3 days: both kidneys presented a longitudinal diameter within the normal centile for the gestational age, measuring 19.3 mm on the left (**A**) and 20.5 mm on the right (**B**).

A 35-year-old mother, gravida 2, para 1, regularly attended the scheduled US scans for the first trimester. At 13 weeks of gestation (wg), the US showed no anomalies, and the nuchal translucency (NT) was of 2.12 mm. The Harmony™ non-invasive prenatal test (NIPT) showed a low risk for trisomies 21, 18, and 13. The patient presented at 21 weeks + 3 days for the planned morphological scan of the second trimester. The US showed oligo/anhydramnios, IUGR (fetal measurements below the 1st centile), a velum interpositum cyst, and mild pericardial effusion. The longitudinal diameter of both kidneys was within the normal centile for the gestational age, the left measuring 19.3 mm and the right, 20.5 mm, respectively (Figure 1) [12].

**Figure 2:**
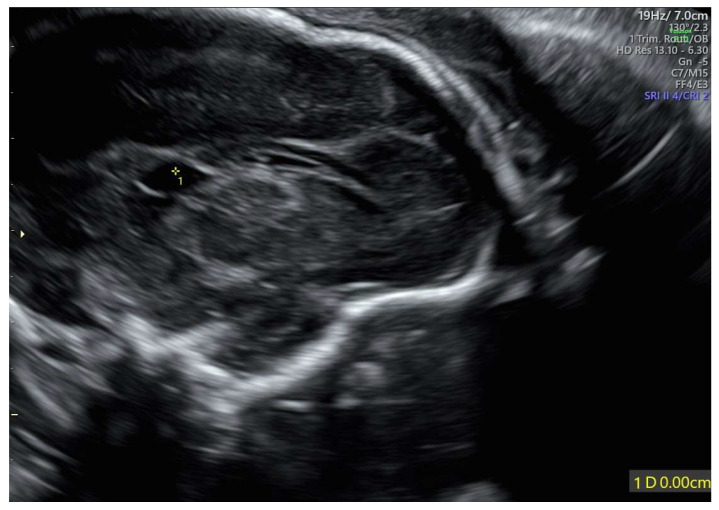
Velum interpositum cyst: the structure (indicated by 1*) was located posteriorly to the corpus callosum.

The velum interpositum cyst was located posteriorly to the corpus callosum (Figure 2).

No other malformations were detected, albeit detection was limited by poor acoustic windows due to low amniotic fluid.

### 2.2. Genetic Investigations

**Figure 3:**
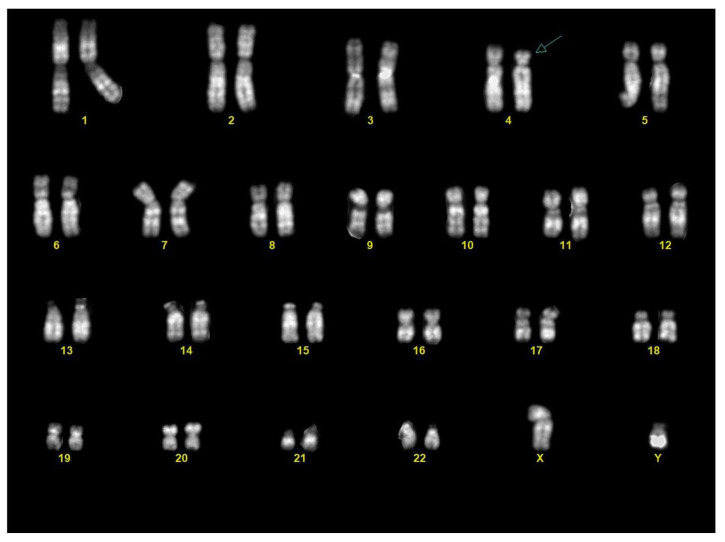
Karyotype from chorionic villi: the karyotype showed a male fetus (46,XY) with a terminal deletion of the distal part of the short arm of chromosome 4 (arrow).

Chorionic villous sampling was performed three days after the US. Karyotype analysis on the chorionic villi revealed a male fetus (46,XY) with a terminal deletion on the short arm of one chromosome 4, from band 4p15.3 (Figure 3).

**Figure 4:**
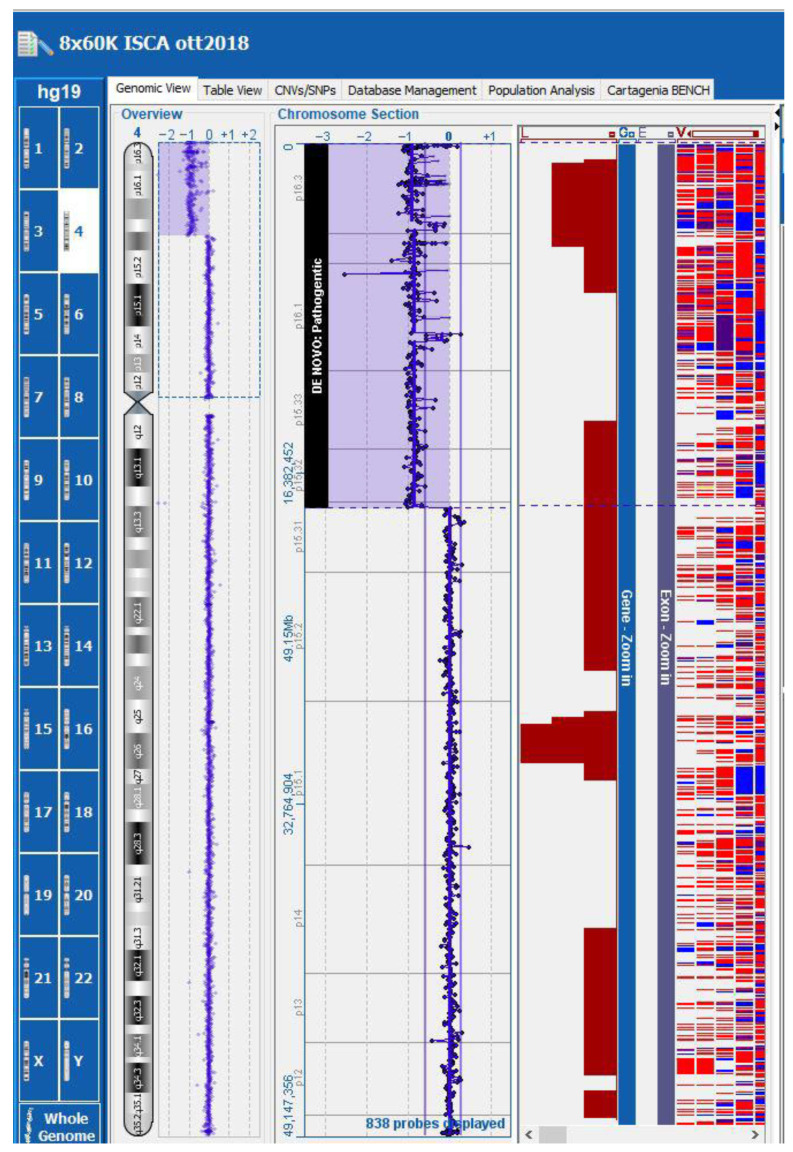
Array-CGH: the fetus presented a de novo terminal deletion of approximately 17.92 Mb, from 4p16.3 to 4p15.31 (genomic coordinates chr4: 37,336–17,953,610 [hg19]).

Subsequent array comparative genomic hybridization (array-CGH) in a trio analysis (chorionic villi and lymphocytes from both parents) identified in the fetus a de novo terminal deletion of approximately 17.92 Mb, from 4p16.3 to 4p15.31 (genomic coordinates chr4: 37,336–17,953,610 [hg19]) (Figure 4). This deletion included the critical region for WHS (WHS; OMIM #194190).

The deletion was confirmed to be absent in both parents through conventional karyotyping and FISH using a WHS-specific probe (4p16.3), which demonstrated normal chromosomal assessment in both. These findings supported the diagnosis of WHS due to a de novo chromosomal rearrangement.

Genetic counseling reported both parents as a non-consanguineous couple. The mother was 35 years old and of South American origin; the father was 40 and Arab. Both partners were in good general health. The mother reported no history of growth issues and had a previous healthy son from a different partner. The male partner also had a son in good health from a previous relationship and two early spontaneous abortions (around the second month of gestation) associated with a maternal infection, though documentation was unavailable. He reported a history of delayed growth during puberty that resolved spontaneously. Family history was reportedly unremarkable for intellectual disability, congenital malformations, or known genetic disorders. However, the male partner reported a 12-year-old nephew (child of his sister) who experienced delayed speech acquisition, with subsequent resolution and no intellectual disability (no records available).

Following genetic counseling, the couple opted for termination of the pregnancy at 23 weeks’ gestation.

### 2.3. Post-Mortem Investigations

Skeletal X-rays showed no evident ossification abnormalities, although evaluation of the phalanges was limited.

**Figure 5:**
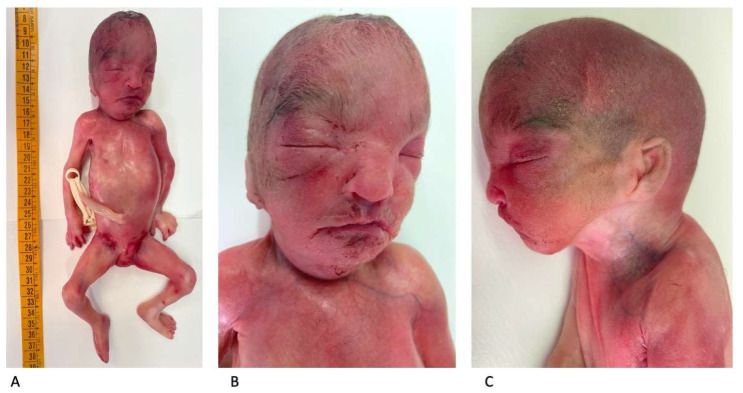
Postmortem external examination: mild fetal growth restriction (crown–heel length: 30.1 cm) (**A**). Facial dysmorphism consistent with WHS (**B**,**C**).

An autopsy showed a fetus with mild growth restriction. The anthropometric measurements were as follows (compared with the normal range for gestational age) [13]: weight 456 g (510 +/−97 g); crown–heel length 30.1 cm (29.1 +/−2 cm); head, thoracic, and abdominal circumference, 19.5 cm (20.7 +/−1.4 cm), 16 cm (17 cm), and 14 cm (15.1 cm), respectively; and foot length 4.1 cm (4.1 +/−0.2 cm). An external examination showed facial dysmorphism compatible with WHS: broad forehead, hypertelorism, flat nasal bridge, deep philtrum, downturned corners of the mouth, microretrognathia, and low-set and posteriorly rotated ears (Figure 5A–C).

**Figure 6:**
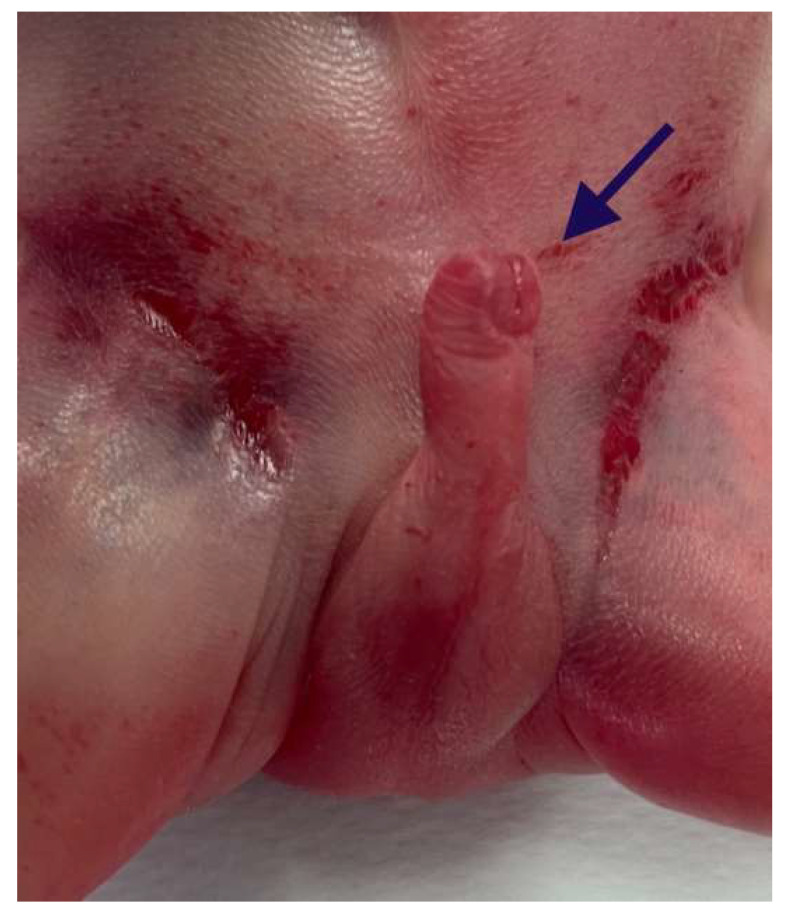
Ventral hypospadias: the urethral meatus was located posteriorly (arrow).

The external genitalia showed ventral hypospadias with a posteriorly located urethral meatus (Figure 6).

The internal examination showed renal hypoplasia, in contrast to the recent fetal US, with small kidneys measuring 12 mm longitudinally. Renal lobation was absent. Both kidneys weighed 0.1 g each (compared to a normal combined weight for the gestational age of 4.77 +/−1.39.) The ureters presented reduced caliber, regularly terminating into the bladder.

The fetal brain displayed primary sulci, such as the Sylvian fissure, parieto-occipital fissure, calcarine sulcus, and cingulate sulcus, with the insula beginning to be covered and early convolutions forming, but secondary and tertiary sulci were not yet present and the cortex remained largely smooth. At slicing, the corpus callosum was complete. The following structures were identified: the septum pellucidum, fornix, and quadrigeminal plate. The deep gray matter nuclei appeared normal, including the basal ganglia (caudate nucleus, claustrum, putamen, and globus pallidus) and their associated fiber tracts (internal, external, and extreme capsules), as well as the thalamus and hypothalamus. The hippocampus was normally convoluted, and the dentate gyrus was identifiable. The ventricular system was patent, including the interventricular foramen of Monro, the cerebral aqueduct (of Sylvius), and the lateral recesses of the fourth ventricle. The origin of the cranial nerves appeared normal, from the first to the twelfth pair. The brainstem showed the olives, pyramids, and decussation anteriorly, and the gracile and cuneate fasciculi posteriorly. The cerebellum was divided into the central vermis and lateral hemispheres. The dentate nucleus was evident on sectioning. The Circle of Willis appeared normal, with visualization of the basilar artery, posterior cerebral artery, posterior communicating artery, middle cerebral (or Sylvian) artery, anterior cerebral artery, and anterior communicating artery. The previously noted interhemispheric cyst was no longer evaluable, possibly due to postmortem collapse.

Regarding the other organs, no abnormalities were identified, except for lung hypoplasia. The lungs’ combined weight was below the expected range for the gestational age (right lung 4.1 g; left lung 3.6; expected for 23 weeks: 13.5 +/−4.4) [13].

The heart was located in its normal anatomical position, with the apex directed to the left and formed by the tip of the left ventricle. The pericardial cavity contained a small pericardial effusion. Atrial situs was solitus. The right atrium showed normal muscular trabeculations and a well-defined crista terminalis. The coronary sinus was in its expected position. The foramen ovale was patent. The left atrium demonstrated smooth internal walls. The atrioventricular connections were normal in configuration and location. The tricuspid and mitral valves had structurally regular leaflets. The pulmonary and aortic valve cusps were morphologically normal. The coronary ostia were in their typical positions. The coronary arteries followed a normal course: the left coronary artery gave rise to the left anterior descending artery and the circumflex branch; the right coronary artery gave rise to the posterior interventricular branch. The systemic and pulmonary venous returns were normal. The pulmonary trunk and ascending aorta were in a normal spatial relationship and had comparable calibers. The ductus arteriosus (ductus of Botallo) was patent. The cervical vessels demonstrated a normal anatomy: on the right, the brachiocephalic (innominate) artery gave rise to the right common carotid artery and the right subclavian artery; on the left, the common carotid artery and subclavian artery originated directly from the aortic arch. The brachiocephalic vein was present inferior to the thymus. The aorta demonstrated normal origin and branching of the intercostal arteries, the celiac trunk, the superior mesenteric artery, the renal arteries, and the inferior mesenteric artery.

**Figure 7:**
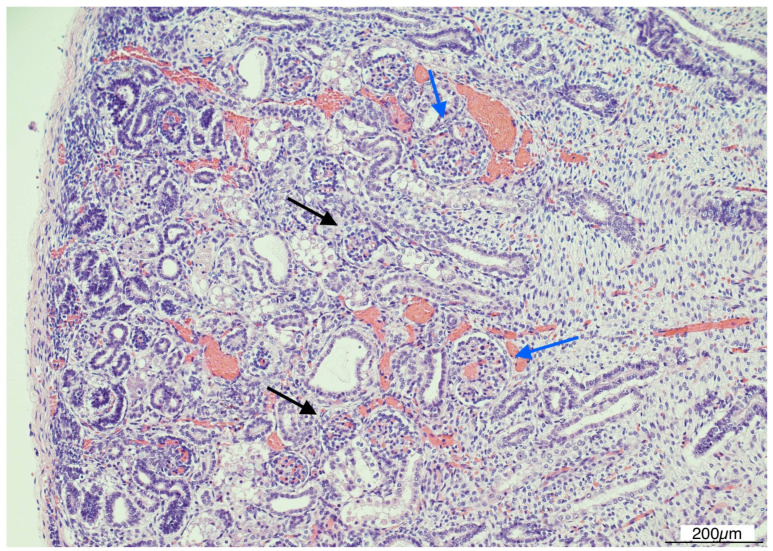
Oligomeganephronia (OMN). Both kidneys presented cortical thinning, and in the medulla, the mesenchymal stroma appeared increased, with a relative reduction in tubules. Glomeruli were overall reduced and hypotrophic (black arrows), as a compensation focal glomerular hypertrophy was present (blue arrows); proximal tubules were also dilated (Hematoxylin and Eosin 10HPF).

Histologically, OMN was the most striking finding in both kidneys. The cortex was thinned, with the glomeruli overall reduced, most of them hypoplastic, but a few hypertrophic. The proximal tubules were enlarged as well. In the medulla the tubules were scattered and intermixed with the mesenchymal stroma (Figure 7).

**Figure 8:**
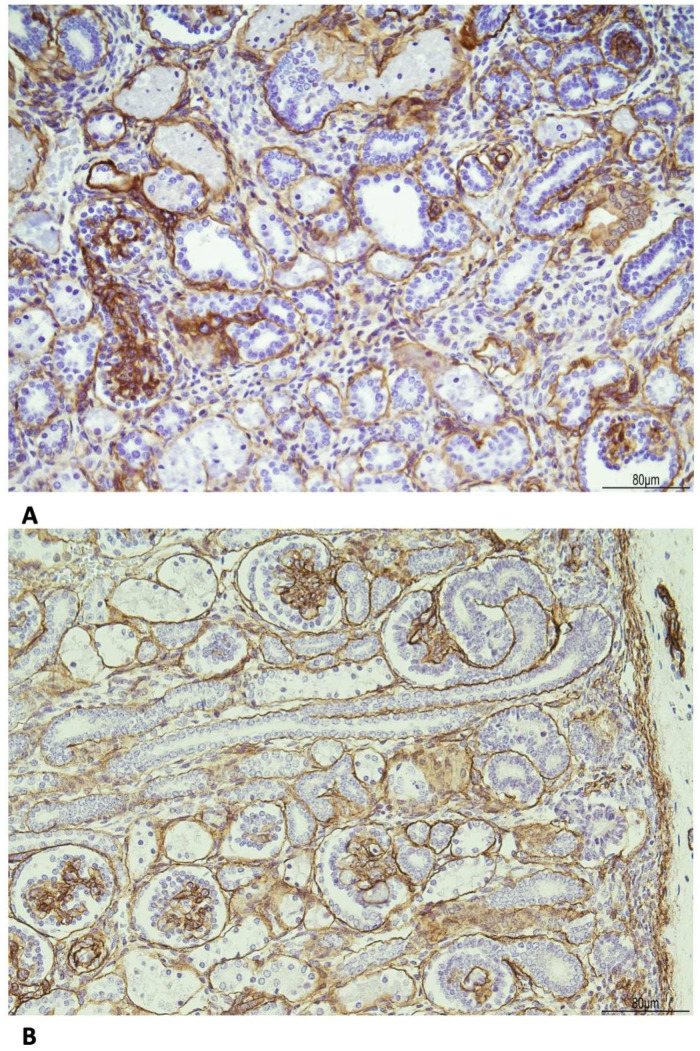
Immunohistochemistry for Collagen IV shows the following: (**A**) WHS: the basal membrane was thickened in the glomeruli and tubules (20 HPF). (**B**) Age control kidney at 23 weeks of gestational age: the basal membrane is overall threadlike (20 HPF).

The basal membrane, especially around the tubules, was thickened as evidenced by the immunohistochemistry for Collagen IV (Figure 8).

**Figure 9:**
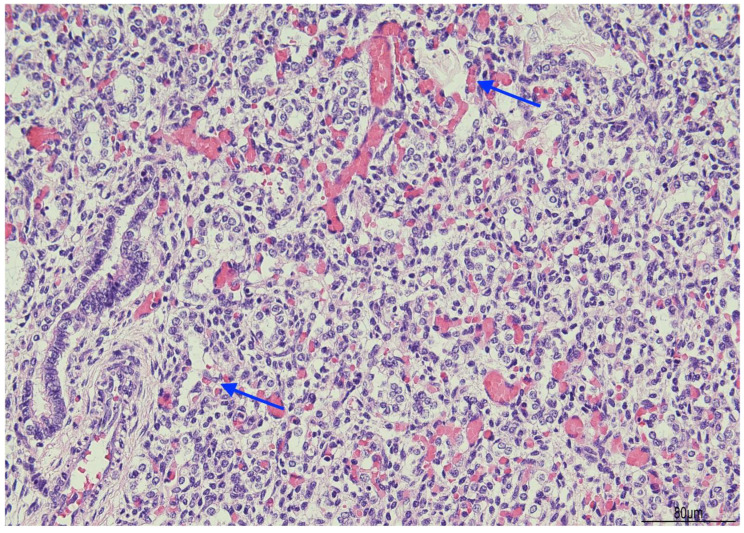
Lungs: early canalicular stage of development. Focal bronchioalveolar duct junctions were formed (arrows), and initial primitive acini had begun to differentiate.

Regarding the other organs, the lungs presented an early canalicular stage of development (Figure 9), slightly delayed for the gestational age [14].

In the brain, neuronal migration was regular, both radial and tangential. The periventricular germinal matrix was abundant. The hippocampus was normally rotated, with preserved architecture of the various sectors of the Cornu Ammonis and the dentate gyrus. The gray nuclei and white matter tracts were unremarkable. The pyramidal tracts, convoluted olivary nuclei, and arcuate nuclei were present. The cerebellum demonstrated all four cortical layers (external granular layer, molecular layer, Purkinje cell layer, and internal granular layer). The dentate nucleus was present.

The myocardium was structurally regular.

**Figure 10:**
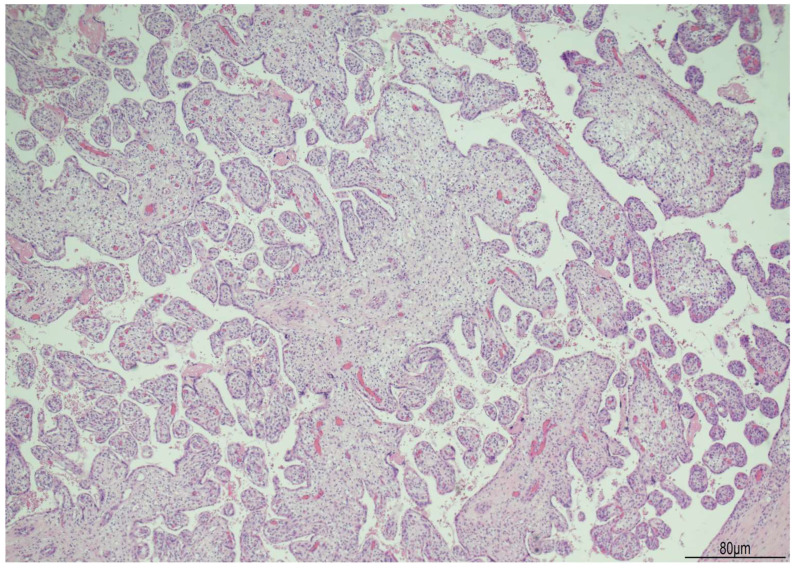
Placenta in WHS: the villi were irregularly shaped and slightly dysplastic (Hematoxylin and Eosin 4HPF).

The placenta weighed 160 g and measured 100 × 9 × 5 cm. The 30 cm cord was hypercoiled with 6 turns every 10 cm. Histologically, the villi were irregularly shaped, bulbous, and with a slightly dysplastic appearance (Figure 10).

## 3. Discussion

WHS, a condition due to a subtelomeric deletion on the short arm of chromosome 4, presents with multiple abnormalities. The typical clinical features include a distinctive “Greek helmet” facial appearance, growth restriction before and after birth, developmental delays, intellectual disability, and seizures [1]. Clinical manifestations correlate with the size of the deletion and can be divided in three groups: (1) deletions under 3.5 Mb result in a mild form; (2) deletions between 5 and 18 Mb are associated with the classic WHS features; (3) large deletions over 22 Mb generally lead to severe malformations [6].

The prenatal US findings are not specific; however, symmetric IUGR, hypoplastic/absent nasal bone, and microcephaly may at least prompt prenatal genetic investigations [1,7,15].

In our case, the US findings, at 21 weeks + 3 days, included IUGR, oligo/anhydramnios, velum interpositum cyst, and mild pericardial effusion. Despite oligo/anhydramnios, both kidneys were not hypoplastic, measuring in longitudinal diameter 19.3 mm on the left, and 20.5 mm on the right, respectively [12].

The array-CGH on the chorionic villi detected a terminal deletion of approximately 17.92 Mb on the short arm of chromosome 4, from the 4p16.3 to 4p15.31 regions. FISH analyses of the 4p region in the parents were negative and confirmed a fetal de novo deletion not related to parental rearrangements. The parents opted for a legal termination of the pregnancy at 23 weeks of gestational age.

At the postmortem, the facial features were consistent with WHS: broad forehead, hypertelorism, flat nasal bridge, deep philtrum, downturned corners of the mouth, microretrognathia, and low-set and posteriorly rotated ears. A mild growth restriction was also evident with a weight of 456 g (normal for gestational age: 510 +/−97 g) and a crown–heel length of 30.1 cm (normal for gestational age: 29.1 +/−2 cm) (13). Externally, ventral hypospadias with a posteriorly located urethral meatus was observed. The internal examination showed severely hypoplastic kidneys, measuring 12 mm in longitudinal diameter and weighing 0.1 g each. Histologically, the most striking feature was bilateral OMN. The cortex appeared thinned, with an overall reduction in glomeruli, most of which were hypoplastic, though a few were hypertrophic. The proximal tubules were also enlarged. In the medulla, the tubules were dispersed and interspersed with the mesenchymal stroma.

In WHS, hypospadias has been related to Muscle Segment Homeobox 1 (*MSX1*) deletion or haploinsufficiency [16]. MSX1 is a key transcriptional repressor expressed on the ventral side of the developing anterior pituitary and is implicated in the differentiation of gonadotrope cells. During embryogenesis, MSX1 negatively regulates pituitary development by repressing the expression of gonadotropin-releasing hormone (GnRH) genes [16].

Genitourinary (GU) anomalies are relatively uncommon in Wolf–Hirschhorn syndrome (WHS). In a comprehensive review of prenatal cases [13], such anomalies were reported in only 23 out of 118 cases (19.5%). Although renal hypoplasia has been frequently mentioned, its exact histological features have rarely been taken into account, even in cases of an interruption of pregnancy. Only in five fetuses and four pediatric cases are the pathological characteristics of OMN better described and related to renal hypoplasia [8,9,10,11].

Renal hypoplasia is generally classified into three main types: segmental hypoplasia (also known as Ask-Upmark kidney), oligomeganephronic hypoplasia (oligomeganephronia), and simple hypoplasia. All types share the common feature of a reduced number of renal lobes. Cortical hypoplasia, characterized by cortical thinning and a shrunken medulla, though not currently categorized, is also included [17].

The literature on OMN is limited, with only a single large case series reported [18]. The clinical diagnostic criteria are much more lenient and they include the following: (1) reduction in the total renal size, defined as the combined length of both kidneys being ≤80% of the expected length of a single kidney in a child of comparable size; (2) decrease in the glomerular filtration rate to 30% of normal values; (3) no evidence of urinary tract malformations or significant vesicoureteral reflux (minor reflux was acceptable if no renal scarring was present) [18]. OMN is typically bilateral, characterized by the symmetrical involvement of both kidneys. At birth, the kidneys are markedly small and exhibit a reduced number of renal lobes, typically five to six or fewer, even one or two [17]. The clinical manifestations typically begin in early childhood with impaired urinary concentrating ability, leading to symptoms such as dehydration, polyuria, and polydipsia. As the disease progresses, hyperfiltration and proteinuria develop, eventually leading to progressive renal failure. The outcome of this failure is influenced by the amount of remaining functional renal tissue. Hypertension is rare [17]. In the series by Broyer et al. [18], end-renal stage disease (ERSD) developed between 6 months and 17 years of age.

Histologically, microdissection studies have shown, in the early stages of the disease, enlargement of the whole nephron, involving the glomeruli, juxtaglomerular apparatus, and tubules. The capillary loops are also increased in number. Due to hypertrophy of the glomeruli and tubules, glomerular density is reduced, with fewer glomeruli per millimeter of cortex compared to age-matched controls. The disease evolution includes segmental and total glomerulosclerosis with tubulointerstitial scarring [17].

The association between renal hypoplasia, OMN, and WHS is very rare, and the details of the cases reported in the literature are summarized in Table 1 [8,9,10,11].

Tachdjian et al. [8] described five fetuses with WHS diagnosed with a standard karyotype. The cytogenetic analysis identified breakpoints within the 4p16 region in three cases, 4p15 in one case, and 4p14 in another. The deletion was de novo in four cases, while in one case, it was associated with a paternal balanced translocation. Four fetuses underwent legal interruption of pregnancy in the third trimester (at 30, 26, 35, and 37 weeks of gestational age, respectively). One case was born at 33 weeks and died after 5 min. All the fetuses and the newborn presented with IUGR and typical craniofacial features. Three fetuses were female and two males, both presenting with hypospadias. Interestingly, post-mortem X-rays disclosed in all cases the verticalization of the nasal bone. Severe renal hypoplasia with histological OMN was present in all of them, and in the newborn, there were also dysplastic cortical areas. Unfortunately, no renal histological pictures were published. Despite renal hypoplasia, the quantity of amniotic fluid was reported as within normal limits. This finding probably reflected kidney compensation. On the contrary, in our case there was oligo/anhydramnios, likely due to impaired kidney function.

Park and Chi [9] reported two infants with severe renal hypoplasia. They died at 4 months and 2 days after birth. The first infant died from kidney failure and the second from respiratory insufficiency. Both infants underwent standard cytogenetic studies revealing the following karyotypes: 46,XY r(4p) and 46,XY del(4p)(p12), respectively. At the autopsy, in both cases, the kidneys were very small and not properly lobated and with an indistinct corticomedullary junction. A thorough morphometric analysis using a microscope revealed a reduced number of glomeruli per square area and glomerular hypertrophy, features compatible with OMN.

Sakallioglu and Gok [10] described a two-year-old child with WHS and a 4p deletion [46, XY, del (4p16.3)] detected by conventional cytogenetic studies. The deletion was found de novo in the patient. The child presented with proteinuria, which was subsequently resolved with angiotensin-converting enzyme inhibitors (ACEIs).

Gatto et al. [11] reported a 9-year-old child with a de novo 3.5 Mb deletion detected by an array-CGH, arr 4p16.3p16.2 (61,552–3,814,895), encompassing the WHSCR-2 region. IUGR was reported in pregnancy, and the phenotypic features included the typical Greek warrior face. The child showed mild cognitive impairment and a slight delay in language development, while motor abilities were normal. The patient was admitted for a follow-up examination, and the urine protein-to-creatinine ratio (UP/UCr) was 4.8 mg/mg (normal values for age <0.5 mg). An abdominal US detected severely hypoplastic kidneys (longitudinal diameter: right 5.5 cm and left 7 cm). The renal biopsy evidenced OMN with focal segmental glomerulosclerosis (FSGS) with concomitant early tubulointerstitial sclerotic atrophy. The patient started the ACE inhibitor (ACEI) Ramipril, progressively improving the UP/UCr-.

OMN can be classified as isolated or associated with congenital anomalies in conditions such as WHS, *PAX2* mutations, and acrorenal syndrome [19]. In WHS, renal involvement can have a neonatal onset, while in acrorenal syndrome, it is usually more subtle, beginning in infancy or later in adolescence. Renal features commonly include proteinuria, high creatinine levels, polyuria, dysuria, and hypertension. These manifestations are the result of progressive overload on the limited number of glomeruli, which increases their volume and causes damage to the glomerular basement membrane. This damage can lead to hypertension, haematuria, proteinuria, and a decline in renal function. Additionally, urinary protein levels gradually rise as renal failure progresses [19]. In WHS, there is no specific association between the extent of the 4p deletion and renal hypoplasia, though in general, the more extensive the 4p deletion, the worse the phenotype [20]. In our case, there was a discrepancy between the US renal findings of dimensionally regular kidneys at 21 weeks + 3 days and the severely hypoplastic kidneys at the postmortem examination at 23 weeks. The US images were reviewed, and no errors in measurements were detected. This finding would interestingly suggest that at 21 weeks + 3 days, the kidneys, although of normal dimensions, were already functionally impaired as the clinical evidence of oligo/anhydramnios shows. In approximately 10 days, the kidneys strikingly regressed, showing OMN at histology.

This feature has never been reported and prenatal diagnosis in cases of WHS must take into account that kidneys, although presenting with normal measurements, in the presence of oligo/anhydramnios, may be already functionally impaired and may further regress.

Currently, there is no definitive cure for OMN, and clinical management focuses on symptomatic treatment. Using antihypertensive agents, particularly angiotensin-converting enzyme (ACE) inhibitors or angiotensin receptor blockers (ARBs), may help to reduce glomerular hyperfiltration and thus slow the progression of the disease. However, monotherapy with ACEIs or ARBs is often insufficient for patients with significant proteinuria and FSGS. In such cases, corticosteroids or statins may be prescribed. Nevertheless, OMN usually progresses to ESRD, for which long-term renal replacement therapy or early kidney transplantation may be more effective treatment options.

## 4. Conclusions

OMN could be the histological hallmark in WHS, and prenatal management must consider that although kidneys may be dimensionally normal, they could shrink in a very short time. Oligo/anhydramnios may be an early marker of renal functional impairment, and strict US follow-up should follow. This association could help to define early prognosis, as well as appropriate clinical management and treatment. However, the connection between renal functional impairment indicating OMN and further renal hypoplasia requires confirmation through further morphological and histological studies.

## Figures and Tables

**Table 1:** Review of the literature of WHS cases with renal hypoplasia and OMN.

Ref.	Age	Cytogenetic	Inheritance of Deletion	Phenotypic Anomalies	Kidneys	Pregnancy	Placenta
[8]	TOP-30 wga	46,XX,del(4)(p14)	De novo	IUGR, dolicocephaly, facial dysmorphism, micro-rerognathia, incomplete pulmonary lobation, gut malrotation, abnormal liver lobation, absent gallbladder, bicornuate uterus. X-rays: nasal bone hypoplasia, delayed bone-age.	Renal hypoplasia: 4 g (combined weight); absent lobation; OMN.	Polyhydramnios	Hydropic villi
[8]	TOP-26 wga	46,XY,del(4)(p16)	De novo	IUGR, facial dysmorphism, microretrognathia, right cleft lip and complete cleft palate, clinodactyly, hypospadias, membranous ventricular septal defect, incomplete lobation of right lung.X-rays: verticalization of nasal bone, delayed bone-age	Renal hypoplasia: 2.8 g (combined weight); absent lobation; OMN.	Normal amniotic fluid	Accelerated villous maturation and fibrin deposits
[8]	Born 33 wga: died 5 min of life	46,XX,del(4)(p15)	Paternal reciprocal translocationt(4;19)(p15;p13)	IUGR, dolicocephaly, facial dysmorphism, microretrognathia, three lobes in both lungs, partial gut malrotation.X-rays: verticalization and hypoplasia of nasal bone, delayed bone-age.	Renal hypoplasia: 3.4 g (combined weight); absent lobation; OMN with focal cortical dysplasia.	Normal amniotic fluid	Multiple infarcts
[8]	TOP-35 wga	46,XX,del(4)(p16)	De novo	IUGR, dolicocephaly, facial dysmorphism, microretrognathia, both lungs with two lobes.	Renal hypoplasia: 7 g (combined weight); absent lobation; OMN with focal cortical dysplasia.	Normal amniotic fluid	No anomalies
[8]	TOP-37 wga	46,XY,del(4)(p16)	De novo	IUGR, dolicocephaly, facial dysmorphism, microretrognathia, hypospadias.X-rays: delayed bone-age.	Renal hypoplasia: 9 g (combined weight); absent lobation;OMN with focal cortical dysplasia.	Normal amniotic fluid	Two-vessel cord, multiple infarcts
[9]	Death at 4 months due to kidney failure	46,XY r(4p)	Not known	Low birth weight, facial dysmorphism, micrognathia, bilateral cleft lip and palate.	Renal hypoplasia: 5.3 g (combined weight); absent lobation; OMN.	Not known	Not known
[9]	Death at 2 days of life due to respiratory insufficiency	46,XY,del(4)(p16)	Paternal translocationt(4:6)(p12,p23)	Low birth weight (full term, 2.1 kg), microcephaly, bilateral cleft lip and palate, hypospadias, bilateral genu varus and equinovarus clubfoot.X-rays: absence of the 11th and 12th ribs, kyphoscoliosis with fusion anomaly of T11 and partial fusion of L2.	Renal hypoplasia: 3.1 g (combined weight); absent lobation; OMN.	Not known	Not known
[10]	2 years old	46, XY, del (4p16.3)	De novo	Facial dysmorphism, micrognathia, proteinuria.	Renal hypoplasia at US, renal biopsy with OMN.	Not known	Not known
[11]	9 years old, 4 years of complete follow-up	a-CGH: 4p16.3p16.2 (61,552–3,814,895) (3.5 Mb deletion)	De novo	Facial dysmorphism, UP/UCr of 4.18 mg/mg at first visit.	Renal hypoplasia at US (longitudinal diameter: right 5.5 cm, left 7 cm; parenchymal thickness right 1.6; left 1.5 cm). Renal biopsy with OMN: focalsegmental glomerulosclerosis with initial tubulointerstitial sclerotic atrophy.	IUGR	Not known
Current case	TOP-23 wga	a-CGH: 4p16.3p15.31 (37,336–17,953,610) (17.92 Mb deletion)	De novo	Facial dysmorphism, ventral hypospadias.	Renal hypoplasia absent at US. Postmortem: renal hypoplasia with kidney longitudinal diameter 1.2 cm; weight: 0.1 g each. OMN.	Oligo/anhydramnios	Dysmorphic/dysplastic villi

Abbreviations: TOP: termination of pregnancy; IUGR: intrauterine growth restriction; OMN: oligomeganephronia; a-CGH: array-CGH; UP/UCr: urine protein-to-creatinine ratio.

## Data Availability

The data presented in this study are available upon request from the corresponding author.
